# Four-drugs regimen containing raltegravir is highly effective in HIV patients starting therapy with >500,000 copies/mL viral load

**DOI:** 10.7448/IAS.17.4.19774

**Published:** 2014-11-02

**Authors:** Gaetana Sterrantino, Mauro Zaccarelli, Francesca Prati, Andrea Boschi, Laura Sighinolfi, Vanni Borghi

**Affiliations:** 1 Tropical and Infectious Diseases Unit, Azienda Ospedaliera-Universitaria Careggi, Florence, Italy; 2 Viral Immunodeficiency Unit, INMI “L Spallanzani” IRCCS, Rome, Italy; 3 Infectious Diseases Unit, IRCCS Santa Maria Bianca, Reggio Emilia, Italy; 4 Infectious Diseases Unit, Azienda Sanitaria di Rimini, Rimini, Italy; 5 Infectious Diseases Unit, Azienda Ospedaliera-Universitaria di Ferrara, Ferrara, Italy; 6 Infectious Diseases Unit, Azienda Ospedaliera-Universitaria di Modena, Modena, Italy

## Abstract

**Introduction:**

Assessing virological response of four-drugs antiretroviral regimen that include raltegravir (RAL) in naïve patients with high viral load (>500,000 copies/mL) selected from a multicentre Italian database.

**Methods:**

Naïve patients with HIV RNA>500,000 copies/mL, who began standard antiretroviral regimens either based on non-nucleoside reverse transcriptase inhibitors (NNRTI) or boosted-PI (PI/r), or a standard regimen plus RAL between 2008 and 2013 were analyzed. Observation was censored at 12 months and the percentage of patients who achieved a viral load below the limit of detection (BLD) was calculated. Virological failure was defined as two consecutive viral loads>40 copies/mL.

**Results:**

Overall, 179 patients were included (13% with primary HIV infection (PHI), and 42.5% with AIDS diagnosis). Of them, 156 started standard three-drugs antiretroviral regimen (75.6% PI/r-based, 24.4% NNRTI-based. Among patients with PHI, 23 patients (12.8%), 6 (25%) started a four-drugs antiretroviral regimen containing both RAL and PI/r. Patients’ characteristics were as follows: males 74%, median age 42 years (IQR 35–51), sexually transmission 75.1%, median CD4 count 156 cells/µL (IQR 47–368) and median HIV-RNA 6.1 log10 copies/mL (IQR 5.8–6.4). 91 of 179 patients (50.8%) reached BLD viral load during the twelve months of observation. Three patients (1.7%) who began regimens PI/r-based with three-drugs had virological rebound after reaching BLD viral load. By use of survival analysis, we show that those patients who added RAL to the standard regimen have reached the primary end point faster (mean 8.4 months (95% CI 7.2–9.6) vs 11.4 (95% CI 11.0–11.8) in PI group and 10.3 (95% CI 9.4–11.1) in NNRTI group; p<0.001, Figure 1). In the adjusted analysis, the choice of a standard regimen versus a four-drugs regimen was driven only by higher baseline viral load (OR. 9.05; 95% CI 2.41–37.41; p=0.001).

**Conclusions:**

Only half of the naïve patients who began antiretroviral therapy having >500,000 copies/mL HIV-RNA had virological success at 12 months. The success was reached faster using the RAL-containing four-drugs regimen, suggesting that strengthening the initial regimen could be an option in patients with very high viral load to improve virological response.

**Figure 1:**
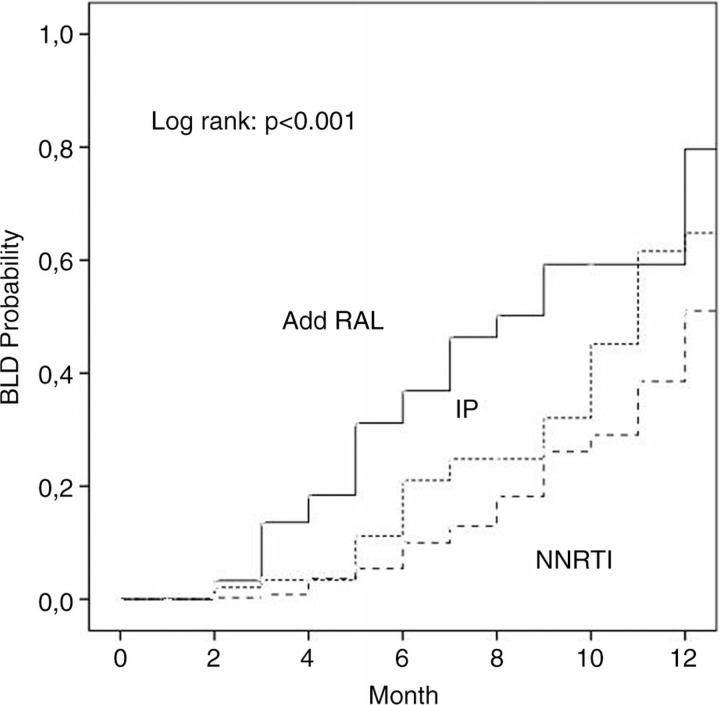
Probability of HIV viral load below the limit of detection in naïve HIV-1 patients with viral loads >500,000 copies/mL treated with a 4-drugs regimen containing raltegravir versus standard therapy with PI/ or NNRTI.

